# Ethical-legal implications of AI-powered healthcare in critical perspective

**DOI:** 10.3389/frai.2025.1619463

**Published:** 2025-07-02

**Authors:** Mohammad Nasir, Kaif Siddiqui, Samreen Ahmed

**Affiliations:** ^1^Faculty of Law, Aligarh Muslim University, Aligarh, India; ^2^NALSAR University of Law, Hyderabad, India

**Keywords:** AI-powered healthcare, AI regulation, augmented intelligence (AI), health data, medical privacy, law and AI

## Abstract

The increasing utilization of Artificial Intelligence (AI) systems in the field of healthcare, from diagnosis to medical decision making and patient care, necessitates identification of its potential benefits, risks and challenges. This requires an appraisal of AI use from a legal and ethical perspective. A review of the existing literature on AI in healthcare available on PubMed, Oxford Academic and Scopus revealed several common concerns regarding the relationship between AI, ethics, and healthcare—(i) the question of data: the choices inherent in collection, analysis, interpretation, and deployment of data inputted to and outputted by AI systems; (ii) the challenges to traditional patient-doctor relationships and long-held assumptions about privacy, identity and autonomy, as well as to the functioning of healthcare institutions. The potential benefits of AI’s application need to be balanced against the legal-ethical issues emanating from its use—bias, consent, access, privacy and cost—to guard against detrimental effects of uncritical AI use. The authors suggest that a legal framework for AI should adopt a critical and grounded perspective—cognizant of the material political realities of AI and its wider impact on more marginalized communities. The largescale utilization of health datasets often without consent, responsibility or accountability, further necessitates regulation in the field of technology design, given the entwined nature of AI research with advancements in wearables and sensor technology. Taking into account the ‘superhuman’ and ‘subhuman’ traits of AI, regulation should aim to encourage the development of AI systems that augment rather than outrightly replace human effort.

## 1 Introduction

The integration of Artificial intelligence (AI) in healthcare has stimulated various advancements in diagnostics, treatment and patient care protocols. The most notable contribution of AI is in making worldclass surgical knowledge and expertise available, in all operation theaters at all times, through an AI-powered conduit “Surgical Collective Consciousness” (Acevedo, 2018). Drawing on population data, the collective renders real-time clinical decision support (Hashimoto et al., 2018). AI has been able to save lives and improve healthcare outcomes by making healthcare accessible and healthcare centers more efficient.

Recently, Med-PaLM, an AI model cleared the US Medical exam with expert level scores. This signals that AI-powered healthcare is the future (Singhal et al., 2023). Nonetheless, the burgeoning technological developments have spurred a range of ethical and legal concerns warranting critical examination (Hanna et al., 2025). This paper examines the questions of choice and responsibility arising with the large-scale collection and use of data by AI. It argues that by prioritizing ethical considerations in the development and deployment of AI, medical professionals can enhance health outcomes and cultivate patient trust, thereby bridging the gap between technological advancements and nuanced healthcare realities (Collins et al., 2024).

Additionally, a robust rights based legal framework is required for balancing privacy interests of patients and AI’s need for their data (Mennella et al., 2024).

## 2 Materials and methods

A comprehensive review of literature the on interaction of AI and healthcare available on medical databases and legal databases including PubMed, Oxford Academic, SCOPUS and reports published by the Indian government institutions and international organizations was conducted. The literature was selected on the basis of its engagement with the legal and ethical issues emanating from AI use in healthcare, with the inclusion criteria focused on interdisciplinary approaches and peer-reviewed work published between 2018 and 2025. A total of 25 research papers were reviewed alongside 06 books, policy documents and relevant government/institutional guidelines. The limitation pertains to the plausible bias in selection of the literature and exclusion of some grey literature.

## 3 Deployment of AI in healthcare

AI is the process of training a computer system using complex and large data sets to build its ability to make decisions and predict outcomes when presented with new data (Floridi, 2023). AI models designed for healthcare rely on the data sets of millions of patients fed into it. For instance, to decide what treatment option is likely to work and which treatment will give the best results for the new patient, an AI model will look at data of past patients having similar medical conditions. Since, every individual has a different set of DNAs and AI models can identify similarities and differences, the genomic data helps AI to make highly informed decisions in respect of diagnosis and treatment (Dias and Torkamani, 2019). This potential of AI has made it a valuable asset. For instance, in cases of cancer which involves complicated diagnostics, AI simplifies the task of identification of the level of cancer and the right treatment plan for doctors. By simply feeding the genomic information gathered from tissue biopsy, reports of blood test results and X-ray images of the liaisons, a doctor can leverage AI models to generate the prognosis and suggest the best treatment option (Pesheva, 2024). A similar tool has been developed in Brisbane and is being used in cancer treatment (Mota et al., 2024). AI has made healthcare more refined than ever, charting a course for highly advanced and accessible precision medicine. It has also emerged as a powerful tool for low-income countries like India. Plagued by the challenges of the “Iron Triangle”—comprising of access, equity and cost—India continues to grapple with resource scarcity and accessibility in healthcare. This has contributed to the suboptimal performance on the latest Healthcare Access and Quality Index, where India is placed at 145 out of 190 countries (Yadavar, 2018). In this context, AI-integration is perceived transformative in Indian healthcare sector. With an adoption rate of approximately 68 percent, AI has significantly advanced preventive care, diagnostics, and personalized medicine. However, more than 92 percent of deployment is still in the proof of concept (POC) stage (NASSCOM, 2024). This stage marks AI’s early development and deployment phase, where the primary objective is to test the ‘feasibility” and effectiveness’ of the technology (AI) in a controlled environment.

Given AI’s promising role in healthcare, it is anticipated to contribute approximately 25–30 billion USD to India’s GDP by 2025 (Press Trust of India, 2025). The market size of AI in Indian Healthcare was 950 million USD and it is likely to reach 1.6. billion by the end of 2025 (Market Size of AI in Healthcare 2025, 2025). Further, the current compound annual growth rate (CAGR) of 22.5% is predicted to expand the market size of healthcare AI in India to 650 billion U.S. dollars by the end of 2025 (Blue Weave Consulting, 2024).

AI has been pivotal in managing one of India’s most prevalent diseases—Tuberculosis. AI technology, specifically Computer Aided Detection (AI-CAD) is widely used to detect TB (Jain et al., 2020). Similarly, Sanjay Gandhi Post Graduate Institute of Medical Sciences, Lucknow, has also developed an AI tool for the rapid detection of tuberculosis with an accuracy of 95% (Nath et al., 2024). These AI-modeled systems address the scarcity of personnel and testing laboratories, making TB care affordable and accessible (Qin et al., 2021; Bhargava et al., 2024). Similarly, in overburdened and understaffed gynecology clinics, AI integration is a gamechanger. It has transformed their approach to patient handling. AI algorithms are used for diagnosis by providing it with patient health information (PHI) and patient history; for curating effective and personalized treatment plans for each patient (Emin et al., 2019). Not only AI has made identification of rare and high-risk cases easier it has also been instrumental in reducing diagnostic errors in OBG (Tanos et al., 2024). Moreover, a critical concern of follow-up and timely medication is addressed by AI, which sends automated reminders to patients and signals the doctors in case of any significant change in the patient’s conditions. This saves the doctors from multiple physical visits to the in-patient wards.

Despite several benefits, AI-powered healthcare has many ethical-legal implications which need prompt consideration (Pham, 2025; Gore and Olawade, 2024).

### 3.1 Implications of AI-use

#### 3.1.1 Privacy concerns in medical AI

The healthcare industry heavily relies on data—PHI including their sensitive personal information (SPI). PHI encompasses demographics (gender, age and marital status), financial information (health insurance), information pertaining to their health, specifically medical procedures, genomics and allied information. Their PHI and PII are collected at many touchpoints digitally and physically, and are exchanged on various platforms from diagnostic centres to hospitals in delivery of healthcare services. In AI-powered healthcare, PHI and PII are provided to the AI system for generating outputs, for instance, genome data is given to AI for identifying a person’s anti-microbial resistance to a set of drugs (Ali T. et al., 2023). Such a use of data makes it both an asset and a potent vulnerability. The management of SPI by AI systems raises various questions relating to privacy and data protection. Moreover, the possibility of re-identification of pseudo-anonymized patient data remains a significant challenge. Further, with commercialisation of medical AI, the risks of exploitation of data, its misuse and breach have also increased.

Our existing regulations and laws are not designed for AI integrated healthcare; but for physical human performed surgeries and treatments. As AI can become more intelligent and suitable over time to the environment in which it is deployed, it is challenging traditionally established understandings of consent and autonomy. For AI-powered healthcare to flourish there is a need for law enabling data sharing and simultaneously protect the patient privacy.

For instance, an Apple Watch uses AI to monitor real-time user’s heart rhythms. This collection of data by AI which it eventually uses to train its algorithm raises severe data security and privacy risks and with sharing for purposes of innovations the risk of intensifies manifolds (Gerke and Rezaeikhonakdar, 2022).

#### 3.1.2 Bias in medical AI

Holdsworth (2023) describes AI Bias as referring to “the occurrence of biased results due to human biases that skew the original training data or AI algorithm—leading to distorted outputs and potentially harmful outcomes.” For fair and unbiased output, the developers must ensure data diversity. Further, such inherent biases may lead to a of adequate healthcare facilities to marginalized communities, further entrenching existing disparities (Eubanks, 2018).

Gianfrancesco et al. (2018) considers missing data and misclassification as major factors resulting in algorithmic bias. Often, certain unmitigable factors contribute to use of skewed data sets. For instance, to develop an AI model for detecting skin cancer, huge data sets of Caucasian patients available as they are more prone to it as compared to Asian patients. This creates an inherent bias in the model, as it is likely to give biased results for Asian patients. To mitigate such issues, the model must be enabled to give “I do not know” as output in case of an Asian patient. With such modifications, AI is more likely to facilitate more personalized healthcare for all.

A classic case of skewed data culminating into erroneous outputs is of IBM Watson’s Oncology AI. The software was proposed as a game changer, however, on application, it delivered unsafe and erroneous results (Frank, 2019). For instance, to a lung cancer patient with heavy bleeding it recommended a treatment which could have proved fatal. The reason for such erroneous outputs was the limitations in training data. The model was trained with data of few hypothetical cases without inclusion of any real cases or clinical inputs. Ultimately, the hospitals had to discontinue using this model. It is suggested that the policy advisors, healthcare professionals, AI developers and patients must together brainstorm and help in developing AI tools which can serve the whole world and not a miniscule minority. Thus, for bias mitigation and ensuring fairness in medical AI, apart from use of diverse data from heterogenous population for training, various other measures—regular audits, inclusive design teams (policy advisors, healthcare professionals, beneficiary patients and developers). It should be ensured that the algorithms are transparent, explainable and adhere to the fairness metrics—like demographic (same outcome across demographics/groups), equal opportunity (true positive rate) and odds (true and false positive rates) (Xu et al., 2022). Further, feedback from AI users, particularly doctors should be taken at regular intervals to further train the model. The developer’s awareness of existing health disparities and the provision for continuous feedback sharing by users would significantly enhance the effectiveness of AI in healthcare.

#### 3.1.3 Unreliability of AI outcomes

Even when AI seemingly is ‘correct’, the outcomes can still be deleterious (Narayanan and Kapoor, 2024) discuss in detail a 1997 study that tried to use AI to predict outcomes of patients with pneumonia and whether it could make better predictions than healthcare workers. The model’s predictions were fairly accurate, but it discovered that having asthma led to a lower risk of complications due to pneumonia. In reality, however, since the training data was based on the hospital’s existing decision-making system, asthmatic patients had a lower risk for complications *because* they were actually sent to the ICU immediately upon arrival. If the model were to be deployed in the hospital, asthmatic patients would now be more likely to be sent home instead of getting proper care, leading to disastrous outcomes for patients.

#### 3.1.4 Unreliability of narrow AI

Even when AI is trained to work on a very narrow scope and scale, it might still be unreliable. Pasquale explains how a ‘narrow AI’ trained to detect polyps might correctly identify a problem polyp that might be missed by a gastroenterologist yet fail to see any other abnormalities that would be easily spotted by a human because of not having adequate training data for *that* specific abnormality. Thus, an ideal system would be an expert working alongside AI assistance. There is immense value in interdisciplinary collaborations between experts (Ali O. et al., 2023).

#### 3.1.5 Inexplicability

Doctors and patients may often need information beyond AI outputs, i.e., about the features, characteristics and assumptions on which the outputs are based or how the weight between artificial neural networks is interpreted (black box). Such questions underscore the inexplicability debate in the context of informed consent, certification of devices and liability. Doctors often grapple with the challenge of balancing transparency against complexity of AI systems.

The four overarching principles of bioethics are beneficence, nonmaleficence, autonomy and justice adapt fairly well to the new legal challenges posed by the AI use in healthcare. However, when placed in contrast with the other recognized principles governing AI, it becomes clear that an additional principle is required—explicability. Explicability is understood as encompassing both the epistemological sense of intelligibility—answering the question “how does it work?”—and in the ethical dimension of intelligibility—answering the question “who is responsible for the for the way it works.” Explicability opens up the “black box” by making the AI system’s decision-making process transparent and understandable (Floridi, 2023).

#### 3.1.6 The question of liability

The determination of liability when AI is used in healthcare is a challenging task for lawmakers and judges ahead—owing to the lack of applicability of traditional legal principles (Jassar et al., 2022). In cases where AI generates an erroneous diagnosis of a patient, the question of liability arises: who will be liable—the doctor, the AI developer or the concerned healthcare institution? Globally, ambiguities surrounding AI—from the ‘black box’ problem, to the autonomous nature of working of AI systems, to the complex roles of stakeholders—have so far led to an absence of proper legal frameworks (Pai et al., 2024).

#### 3.1.7 The limitations of computer vision

The strengths of AI may in many cases also serve as its weaknesses, owing to how AI technologies essentially work with data and statistical analysis. Hence, while that working could provide insights that humans never could, it could also fail to make the most obvious observations. This is what (Pasquale, 2020) describes when he talks about how ‘computer vision’ can be both superhuman and subhuman at the same time. ‘Superhuman’ here refers to capabilities of carrying out computation on scales much greater than the human brain ever could, while ‘subhuman’ refers to the possibilities of failing at the smallest of tasks that a human child could do easily (Pasquale, 2020). Even the idea of a task completed ‘successfully’ can have multiple interpretations based on the kind of AI system being observed and what the system has been trained to identify as ‘success’. Often, AI systems trained in environments with fixed parameters such as fail in open ended environments (Marcus and Davis, 2019).

### 3.2 Long term impacts of AI in healthcare

#### 3.2.1 Impacts of algorithms

An excessive reliance on algorithms may prove to be detrimental to the healthcare system. Lending too much credence to AI outputs—be they predictions and forecasts or generative content, is harmful to knowledge production and paves way for real-world complexity to get flattened. However, it also has another effect—that of devaluing the very real human creativity and flexibility that is an important part of administrative operations, not just in healthcare but in all other fields. Algorithms and AI ‘encode’ rules, norms and laws into strict code that often cannot be bent or broken in situations where required. Encoded rules in this way have none of the ambiguity and openness of interpretation of traditional legal language (Hassan and De Filippi, 2017). AI also is likely to have impacts on the academic work in healthcare—where unethical use of AI may be detrimental to the cultivation of academic rigor and creativity (Bhargava et al., 2024). Proper frameworks should be developed at the institutional level to deal with issues such as listing AI as co-authors.

#### 3.2.2 Impact on everyday decision making

As (Blasimme and Vayena, 2020) describe, healthcare operators everyday take decisions where they “calibrate objective criteria with the reality of each individual case,”—such as when deciding which patient is to be treated first. The reality of each individual case takes precedence over the usual “objective” order of things. However, it is unlikely that AI-based patient care systems can be programmed to display such flexibility. Many scholars consider hypothetical situations and discuss how AI systems used to calculate the risks of longer stays in hospital admission decisions might end up discriminating against more vulnerable patients. Probably, it is impossible to develop a one-size-fits-all approach to medical AI—and hence, all sorts of rules and legal frameworks would need to be contextually informed (Evans, 2023).

#### 3.2.3 Impact on doctor-patient relationships

Automated systems employment raises ethical concerns within the doctor-patient relationship. With advancement of AI chatbots, their impacts on this relationship are becoming more pertinent. With increasing access to advanced technology, both doctors and patients have become susceptible to misinformation, necessitating a shift in doctor’s skills and roles (Alrassi et al., 2021). Moreover, privacy and data protection issues arise as healthcare data becomes accessible to third parties, warranting stricter regulations (Naithani, 2024). Sauerbrei et al. (2023) emphasize that concrete steps are needed to ensure AI tools positively impact doctor-patient relationships, focusing on empathy, trust, shared decision making and compassion.

#### 3.2.4 Impact on elderly patients

While, in one way, chatbots and AI assistants can ease up the workloads of doctors and caregivers, they can also have a negative, isolating impact on patients who grow more distant from human care and empathy. This is an ethical issue particularly pronounced in the case of elderly patients, where AI can provide care and companionship yet become an unhealthy replacement for actual human interaction (Pasquale, 2020).

## 4 AI and human augmentation

Considering the power differentials at play in the working of AI, vulnerable and marginalized communities are likely to face more harm as a result of the uncritical deployment of AI. This necessitates human-centric deployment of AI (Sigfrids et al., 2023). Pasquale (2020) suggests looking at AI as ‘augmented intelligence’ i.e. as an intelligent aid to a human doctor instead of the more common idea of an ‘Artificial Intelligence’ working autonomously, without human intervention. Serving as a potent tool in clinician’s hands, augmented intelligence strengthens the expertise of clinicians, augments their ability to recognize patterns, make informed decisions and provide effective patient care based on the data-driven insights (Kellogg and Sadeh-Sharvit, 2022). Simply put, it envisions doctors and AI working in conjunction by task-sharing. For instance, ChestLink, an AI model developed by Oxypit is used for triaging (Fanni et al., 2023). It scans the X-rays to identify abnormalities and generates the diagnosis. In case it does not find any, the AI model sends it to a human radiologist (Tables 1, 2).

**Table 1:** Literature landscape: references, themes and conclusion.

References	Themes covered	Conclusion
Ali O. et al. (2023) and Ali T. et al. (2023)	Data integration, bias, privacy issues, patient safety, other legal issues	Discusses the benefits, challenges, methodologies, and functionalities of AI in healthcare. Gives a very detailed review of AI-powered healthcare. Identifies key legal and ethical challenges.
Alrassi et al. (2021)	Augmented AI, AI-human relationship, Doctor-patient relationship	Emphasizes that technology can only augment human expertise, cannot replace it. Underscores the importance of humanistic characteristics and behaviors—critical human skills in healthcare delivery.
Bhargava et al. (2024)	Principles of accountability, data quality, accessibility, equity, validity, and inclusiveness	Critically analyses AI use in biomedical research and publication to highlight the potential challenges. Advocates ethical use of AI and disclosure of AI use in publications.
Blasimme and Vayena (2020)	Informed consent, impact of automation on professional caregivers and patients; fairness, equity and social justice in AI-driven health Clinical AI Oversight Bodies, Disease Surveillance, Health Promotion	Argues that the current legal and regulatory framework does not address the ethical issues arising from AI use. Advances a governance blueprint based on six principles and offers suggestions in the fields of research and patient care.
Chauhan et al. (2022)	Data Privacy	Human touch of a doctor as integral to healthcare. Viewing AI not as a competitor but rather buddy who can reduce human’s workload.
Eubanks (2018)	Unreliability of AI decisions without human validation, Bias induced, Automation, social welfare, algorithmic bias, digital inequality	Explores the relationship between artificial intelligence systems, urban poor and public welfare systems, especially looking at how automated systems drive inequality instead of lessening.
Evans (2023)	Medical privacy, data privacy, AI Bias, Right to Explanation	Critiques the idea of a possible general rule framework that covers all of AI, instead suggests looking into contextually informed and appropriate perspectives. Calls for extending privacy laws to medical AI. Explores the US legal framework and puts it in contrast with EU’s.
Floridi (2023)	AI bias, Ethical principles in AI use	Argues that the core principles of bioethics apply to the challenges of AI with the addition of explicability. Explains the relevance of explicability in soliciting informed consent.
Frank (2019)	Erronoeus Output, Unsafe treatment suggested by medical AI	Explores the tort law remedies to locate the liability in case of injury by AI machines.
Gerke and Rezaeikhonakdar (2022)	Risks of real time health data collection, big data regulation, health data protection, consumer protection	Analyses privacy threats emanating from health apps on mobile phones that utilize AI and ML software and collect data. Highlights the loopholes in the US legal framework and suggests federal laws as appropriate mechanism.
Gianfrancesco et al. (2018)	Over-reliance on AI, biases (missing data, sample size, and underestimation), Algorithmic bias, socio-economic disparities and inaccessibility to healthcare	Proposes diversity in training data as a mitigation strategy. Suggests alleviating social dispairties by thoughtful use of AI in healthcare.
Hassan and De Filippi (2017)	Impacts of Agorithm	Algorithms and AI ‘encode’ rules, norms and laws into strict code that often cannot be bent or broken in situations where required. Encoding removes ambiguity of language.
Jain et al. (2020)	AI-powered healthcare Challenges in AI-integration in Indian healthcare Human confirmation of AI diagnosis	AI can be leveraged to eliminate TB from India. Discusses the challenge of retrieving, digitizing and entering data in digital format. AI generated diagnosis must be approved by a doctor.
Jassar et al. (2022)	3 levels of AI involvement AI’s black box nature Increasing independent agency Liability	Minimal, Intermediate and High Meticulously compares EU and Canadian legal framework on AI. Explores legal principles that apply to AI. Discusses the application of negligence, vicarious liability, strict liability, product liability principles to AI.
Kellogg and Sadeh-Sharvit (2022)	Consent, data security, liability and accountability in AI-assisted mental healthcare	Recommends a collaborative approach involving multiple stakeholders—clinicians, technologists, and ethicists to develop user-friendly and ethically sound effective AI tools. Suggests continuous training and evaluation of the AI models.
Al Kuwaiti et al. (2023)	Failure of AI to show human empathy Technical, ethical, and governance challenges of healthcare AI	Recommends that AI development should align with people’s interests. Emphasizes the need for ethical and legal framework to address ethical, social and technical challenges.
Marcus and Davis (2019)	Limitations of Computer Vision	Describes the narrowness of current artificial intelligence. Asserts that AI works only for particular tasks it is programmed for. Argues that a computer beating a human in Jeopardy! Is a far from reality.
Mota et al. (2024)	Deployment of AI in healthcare. AI-enabled cancer care	Through a survey-based study proves that AI-assisted cancer contouring helps in overcoming many limitations of the current clinical workflow in oncology and facilitates patient-specific therapy selection and treatment planning. Suggests further validation of AI tools.
Naithani (2024)	Patient privacy and confidentiality in the doctor-patient relationship. Increasing access of patient information to third party. Transparency and informed consent in sharing of health data.	Comprehensive analysis of the Indian data protection law and the preceding developments. Argues for higher protection for health data as it is sensitive personal data.
Narayanan and Kapoor (2024)	Deleterious nature of AI Over-arching presence of AI	Uses case study method to explain the perils of AI use and demonstrate its susceptibility to diagnostic errors.
Nath et al. (2024)	AI-powered TB care	Explores the potential of AI-powered software to treat TB. Argues it as a potent tool for TB elimination from India.
Gore and Olawade (2024)	Data quality, interpretability, bias, and regulatory frameworks. Recommendations emphasize the need for robust ethical and legal frameworks, human-AI collaboration.	Focuses on the need for responsible AI. Recommends the need for robust ethical and legal frameworks. Proposes Human-AI collaboration for optimum utilization of AI.
Pai et al. (2024)	Liability for erroneous AI decisions	Engages with the critical question, “who bears the liability when AI-driven medical decisions go wrong.” Considers Black Box, autonomous nature of AI and multifaceted nature of stakeholders as impeding AI legislations worldwide. Compares the relevant directives and guidelines implemented in EU and USA.
Pasquale (2020)	Unreliability of AI, Super-human and sub-human nature of AI, Limitations of Computer Vision AI and Human Augmentation	Explains the unreliability of AI and narrow AI through examples. Suggests human-centric deployment of AI in healthcare. AI only as an intelligent aid to humans.
Pham (2025)	Ethical-legal implications of AI-powered healthcare (bias, accountability, transparency, fairness). Absence of regulatory soft/hard law and policy framework	Suggests inclusive data collection, conscious algorithm development and AI design as measures to mitigate ethical-legal problems of AI use.
Qin et al. (2021)	Deployment of AI in healthcare, particularly in TB care	Furnishes an extensive overview of available CAD products used to interpret CXR images for TB. Creates an open access database of AI-powered TB care tools.
Sauerbrei et al. (2023)	Impact of artificial intelligence on the doctor-patient relationship Black-box issue, Transparency	Analyses the impact of AI in healthcare through a systematic review of literature. Suggests Explainable AI for forging trust. AI-integration as pivot for shared decision-making and increasing patient autonomy.
Shah and Chircu (2018)	Technology design, system accuracy, security, data collection and management and privacy protection	Considers robust laws and regulations as pivots for success of AI in healthcare. Gives key insights on AI-powered wearables and connectivity, disease detection and treatment, patient care, and sensor networks.
Sigfrids et al. (2023)	Critical role of mutual trust, transparency and communication in developing human-centric AI	Proposes expansion of human centred AI to community- and society centered AI approach. Advocates for ethical, sustainable, and trustworthy AI.
Surendran (2024)	Regulation of AI use in academic public health research	Proposes multifaceted approach, extensive training initiatives, ethical policy adaptation to facilitate responsible AI use and a call for researchers to act as self-checks, while using AI in research.
Vollmer et al. (2020)	Transparency, reproducibility, ethics, and effectiveness of AI Using bigger sample size of data for training	Raises 20 critical questions pertaining to transparency, reproducibility, ethics, and effectiveness (TREE). Suggests that all healthcare professionals using AI must ask these questions for safe and effective AI use.
Xu et al. (2022)	Fairness Metrics, Algorithmic bias mitigation methods in medical AI	Discusses at length algorithmic bias, fairness quantification metrics, and bias mitigation methods and provides a ready reckoner of software libraries and tools useful for bias evaluation and mitigation.
Yadav et al. (2023)	Data sharing, Double-edged sword nature of digital healthcare, ethical issues in AI use, AI Bias	Critically evaluates AI driven healthcare. Highlights data sharing, triangulation and ethical issues as emanating from heterogeneity of data representation. Analyses the consequentialist and deontological impacts of data breach. Suggests federated learning, differential privacy, and cryptographic techniques as safety measures. Fails to distinguish the structural difference in Indian and US legal system.

**Table 2:** Summary of key themes and implications of AI use in healthcare.

Main Theme	Sub Theme	Key point
**Deployment of AI in healthcare**	–	Diagnostics, TB care, surgical care. Increased access, equity and affordability in healthcare. Enhanced clinical and diagnostic accuracy and healthcare outcomes. Improved precision medicine.
Implications of AI use	Privacy concerns in medical AI	Risk of breach of sensitive personal data, ethical concerns of consent and data confidentiality.
	Bias in medical AI	Disparities in results and performance of AI due to biased training data, impacting diagnosis and treatment.
	Unreliability of AI outcomes	Erroneous and often inconsistent output owing to the skewed training data. Over-reliance or complete reliance on AI is not recommended.
	Unreliability of narrow AI	The AI trained to perform only specific tasks cannot be used generally. Lack of flexibility in real-world clinical setup.
	Inexplicability	Difficulties in interpreting and understanding the functioning of AI systems, particularly the mechanism of data processing.
	Question of liability	Undefined liability in case of erroneous AI based decisions used in treatment/diagnosis. Who is to be held liable? Human or AI model.
	Limitations of computer vision	Sensitivity to the quality and content of images, Lack of flexibility to work in open environments.
**Long term impacts of AI**	Impact of algorithms	An excessive reliance on algorithms may prove to be detrimental to the healthcare system.
	Impact on everyday decision making	Has the potential to reduce human creativity, oversight and expertise if clinicians rely too much on AI.
	Impact on doctor-patient relationship	More prone to ethical concerns, particularly data breaches. Susceptibility to misinformation for both doctor and patient. Challenges in adoption of technology.
	Impact on elderly patients	Lack of human-like empathy, risk of non-human touch to care. AI could isolate patients from human caregivers.
**AI and human augmentation**	–	Enhancing human skills and capabilities with AI tools, instead of completely replacing practitioners.

## 5 Legal and regulatory framework

### 5.1 International regulatory and policy developments

The scope and scale of the data being collected requires researchers to formalize new “best practices” and promote transparency, replicability and an engagement with ethical concern (Vollmer et al., 2020; Surendran, 2024). Given the entwined nature of AI research with advancements in wearables and sensor technology, questions of technology design also need examination (Shah and Chircu, 2018).

Taking cognizance of the legal-ethical issues resulting from AI use in healthcare, the (World Health Organization Guidance, 2021) released containing six guiding principles: the protection of human autonomy, the promotion of human well-being, safety and public interest, ensuring transparency, explainability, and intelligibility, fostering responsibility and accountability, ensuring inclusiveness and equity, and promotion of sustainability. Subsequently it acknowledged large language models (LLMs) as deleterious to patient’s safety, highlighting the dangers of biased data, the illusion of authoritativeness and plausibility in LLM outputs that masks potentially serious errors, the non-consensual data reuse and deliberate disinformation.

World Health Organization (2024) Guidance on AI ethics and governance for LMM AI models reiterates the importance of quality data, warns against ‘automation bias’ and potential cybersecurity risks, advocates for a multi-stakeholder approach, and recommends governments to invest in public infrastructure, draft law and regulation to enshrine ethical obligations and human rights standards, and to establish regulatory agencies for governing AI in medicine.

### 5.2 Regulatory and policy frameworks in India

The Indian model exemplifies a policy-driven, hybrid framework characterized by the interplay of AI governance Advisories, Strategies, (Indian Council of Medical Research, 2023) Guidelines and scattered sectoral Regulations and evolving data protection law, albeit snail paced. The data protection law, i.e., Digital Personal Data Protection Act enacted in 2023 is yet to be enforced. This law permits processing of personal data with consent of the data principal (individual whose personal data is collected) and without consent in certain legitimate cases as outlined in Section 7. The legitimate uses include scenarios where data is required to respond to any medical emergency involving a threat to the life or immediate threat to the health of the person whose data is concerned (data principal) or any other individual; or where it is necessary for taking measures to provide medical treatment or health services to any individual during an epidemic, outbreak of disease, or any other threat to public health.

The Act, however, does not define “health data” or “sensitive personal data” as opposed to its precursors, which creates a regulatory gap, particularly in sectors like health. Moreover, it treats does not enhanced safeguards for sensitive data. This absence of safeguards and clear definition, raises concerns about AI’s potential to exacerbate systemic risks. Without adequate safeguards, AI-driven healthcare may lead to erroneous decisions or exclusionary practices driven by algorithmic bias or non-causal correlations. The importance of addressing regulatory gaps in India cannot be overstated.

Unfortunately, DISHA Act which proposed a rights-based framework for patient’s privacy and enabled digital sharing of PHI between hospitals and clinics, could not see the light of the day and hence, we still do not have an adequate legal framework. Thus, proper legal and ethical frameworks—covering a wide range of issues from consent to access, from privacy to cost, in addition to effective governance mechanisms need to be evolved to protect society from the harmful effects of an uncritical use of AI (Table 3; Al Kuwaiti et al., 2023).

**Table 3:** Regulating AI and data: US, EU and India.

Aspect	United States	European Union	India
Regulatory Model	Decentralized, sector-specific	Unified, structured and risk-based classification of regulations and compliance requirements.	Strategy/Advisory-led framework.
Legal Framework	Health Insurance Portability and Accountability Act, 1996	General Data Protection Regulation, 2018 and Artificial Intelligence Act, 2024.	No specific law. Digital Personal Data Protection Act, 2023. MeitY Advisory: Due diligence by Intermediaries/Platforms under the Information. Technology Act, 2000 and Information Technology (Intermediary Guidelines and Digital Media Ethics Code) Rules, 2021.
Innovation	Flexibility for innovation	Moderate flexibility. Requires extensive documentation, regulatory sandboxes.	Flexible ecosystem for Startups.
Ethical oversight	Voluntary (NIST AI Risk Management Framework) Varies across sectors	Focuses on protection of Fundamental Rights against AI use. Legal framework includes ethical principles.	Ethical guidelines for application of Artificial Intelligence in Biomedical Research and Healthcare by Indian Council of Medical Research.
Implementation agencies	Federal and State agencies FDA Regulates AI and ML powered healthcare devices.	European AI Office.	No dedicated agency.
Implementation status	Ongoing	Phased implementation.	Nascent DPDP Act: not enforced.

## 6 Conclusion

AI use in healthcare is at a nascent stage, yet it has transformed healthcare by enhancing accessibility and outcomes to a large extent. AI-powered healthcare has proved to be a viable solution in hospital settings with less resources but huge patient footfall. A major concern spurred by AI use is patient privacy as AI systems require their health and other sensitive data to function effectively. The increasing incursions into the privacy of human beings are alarming. Consequently, the patients are concerned about data security and its potential breaches, which in turn undermines their trust. AI systems are also likely to violate privacy laws if not handled with care. Moreover, the reliability of AI is often questioned leading to ethical dilemmas pertaining to accountability in situations where the AI-powered systems give erroneous diagnostics. Legal and medical professionals have called for defined guidelines to address the accountability conundrum. On the counts of AI as a replacement of human doctors, it is clear that the humane touch of a doctor in a patient’s treatment is irreplaceable. AI is merely a competent assistant and not an “intelligent competitor” of a human being. Furthermore, inherent bias in AI makes it clear that AI generated output will require verification by a human doctor as complete reliance on AI is not feasible.

Additionally, AI should be deployed consciously because it may and has resulted in widening the disparities in certain situations. Thus, a robust regulatory framework complementing the ethical guidelines are indispensable for navigating the present and anticipated challenges.
